# Rituximab potentially improves clinical outcomes of CAR-T therapy for r/r B-ALL via sensitizing leukemia cells to CAR-T-mediated cytotoxicity and reducing CAR-T exhaustion

**DOI:** 10.1007/s13402-024-00945-7

**Published:** 2024-04-25

**Authors:** Yangzi Li, Qingya Cui, Sining Liu, Lingling Liu, Megyn Li, Jun Gao, Zheng Li, Wei Cui, Xiaming Zhu, Liqing Kang, Lei Yu, Depei Wu, Xiaowen Tang

**Affiliations:** 1 National Clinical Research Center for Hematologic Diseases, Jiangsu Institute of Hematology, The First Affiliated Hospital of Soochow University, Suzhou, 215006 China; 2 Institute of Blood and Marrow Transplantation, Collaborative Innovation Center of Hematology, Soochow University, Suzhou, China; 3 East China Normal University, Shanghai, China; 4 Shanghai Unicar-Therapy Bio-medicine Technology Co., Ltd, Shanghai, China

**Keywords:** Chimeric antigen receptor T cell, Rituximab, r/r B-ALL

## Abstract

**Purpose:**

Despite chimeric antigen receptor (CAR) T-cell therapy has achieved great advances in recent year, approximately 50% of relapsed/refractory B cell acute lymphoblastic leukemia (r/r B-ALL) patients treated with CAR-T experience relapse 6 months post CAR-T treatment. CD20 express on 30 to 50% of B-ALL, which makes CD20 Monoclonal Antibody as one of the potential therapy strategies to decrease the tumor burden and improve the efficacy of CAR-T therapy. Adding Rituximab to chemotherapy protocol had been demonstrated to improve the outcome for CD20-positive ALL. However, rare study explored the influence of Rituximab combined with CAR-T therapy.

**Methods:**

We retrospectively analyzed 20 r/r B-ALL patients who received CAR-T therapy, all of whom had failed multiple lines of therapy. Before CAR-T infusion, we administered Rituximab to 10 patients with high CD20 expression at a dose of 375 mg/m^2^ for 1 day. Meanwhile, we selected 10 patients with the comparable features who underwent CAR-T treatment without Rituximab in the same period as the control group. In vitro, the surface molecule expression and killing of CAR-T post Rituximab-treated B-ALL cells co-incubated with CAR-T cells were detected by flow cytometry.

**Results:**

The median follow-up of Rituximab and Control groups were 29.27 and 9.83 months. We found that adding Rituximab may confer a favorable prognosis compared with Control group. The 2-year overall survival (OS) and leukemia-free survival (LFS) rates both were longer in the Rituximab group (90% vs. 26.7%, *p* = 0.0342; 41.7% vs. 25%, *p* = 0.308). In vitro, we observed that Rituximab-treated tumour cells are more sensitive to CAR-T killing and a broad range of cytokines and chemokines were produced when Rituximab-treated Nalm-6 cells co-cultured with 19-22CAR-T cells, such as interferon-γ (IFN-γ), tumor necrosis factor-α (TNF-α) and interleukin-2 (IL-2). To investigate whether Rituximab has an effect on CAR-T persistence, we stimulated CAR-T cells repeatedly in vitro with Rituximab-treated Nalm-6 to evaluate the changes in CAR-T surface exhaustion molecules at different times. We found that the expression of exhaustion molecules (LAG-3, PD-1, TIM-3) on CAR-T cells were significantly lower in the Rituximab group than in the Control group.

**Conclusion:**

Rituximab combined with CAR-T therapy is effective for improving the long-term prognosis of B-ALL patients who have failed multiple lines of therapy. In vitro, we observed that rituximab potentially improves CAR-T efficacy by sensitizing ALL to CART-mediated cytotoxicity and reducing CAR-T exhaustion.

**Supplementary Information:**

The online version contains supplementary material available at 10.1007/s13402-024-00945-7.

## Introduction

Despite chimeric antigen receptor (CAR) T-cell therapy has achieved great advances in recent year, approximately 50% of relapsed/refractory B cell acute lymphoblastic leukemia (r/r B-ALL) patients treated with CAR-T experience relapse 6 months post CAR-T treatment. Clinical factors such as high disease burden and CAR-T cell exhaustion have been reported to increase the risk of recurrence. Additionally, high disease burden was also accompanied by a markedly higher incidence of cytokine release syndrome [1]. Hence, mitigating pre-CAR disease burden and CAR-T cell exhaustion may represent a crucial and viable strategy for augmenting the effectiveness and safety of CAR-T therapy.

CD20 express on 30–50% of B-ALL [2–5], making CD20 monoclonal antibody a potential therapeutic strategy for reducing tumor burden and enhancing the efficacy of CAR-T therapy. Intravenously administered rituximab was the first therapeutic mAb (Monoclonal Antibody) to be used in the field of oncology, establishing a new class of antitumor drugs. Adding rituximab to chemotherapy protocols has been shown to improve outcomes in CD20-positive ALL. In preclinical studies, rituximab augmented CD19 chimeric antigen receptor (CAR) T-cell function and increased tumor reduction and survival in murine models through synergistic targeting with CAR T-cells. However, few studies have explored how rituximab improves CAR-T efficacy. Therefore, we evaluated the efficacy and safety of rituximab administration before CAR T therapy in relapsed or refractory B-ALL and investigated the influence of rituximab on CAR-T cells as well as B-ALL cells in vitro.

## Methods

### Study design and patient population

We retrospectively analyzed 10 r/r B-ALL patients who received CAR-T therapy at the First Affiliated Hospital of Soochow University between May 2017 and September 2022, all of whom had failed multiple lines of therapy and had high CD20 expression. Before CAR-T infusion, we administered Rituximab to all patient at a dose of 375 mg/m^2^ for 1 day. Meanwhile, we selected the patients with the comparable features who did not receive Rituximab treatment during the same period as the control group. We matched the control group with the Rituximab group at a ratio of 1:1 based on: (1) age; (2) sex; (3) molecular mutations; (4) cytogenetic abnormalities; (5) CAR-T type; (6) conditioning regimen; (7) donor type. The study regimen was approved by the Ethics Committee of the First Affiliated Hospital of Soochow University, and all patients provided written informed consent.

### Manufacture of tandem CD19/CD22 CAR-T cells

CAR-T cells were provided by Shanghai Unicar-Therapy Bio-medicine Technology Co. Ltd. The manufacture of CD19/CD22 CAR-T cells mainly consisted of retroviral vector (mCD19-mCD22 scFv.CD28/4-1BB/z) production, T cell transduction, and CAR-T cell expansion (Fig. 3A). CAR constructs (mCD19-mCD22) were synthesized and cloned into a third-generation lentiviral plasmid backbone. The CAR constructs contained a CD8 transmembrane domain in tandem with CD28, OX40, and CD3ζ transmembrane domain. CAR-T cells were successfully manufactured, and qualified tests were conducted before infusion.

**Fig. 1:**
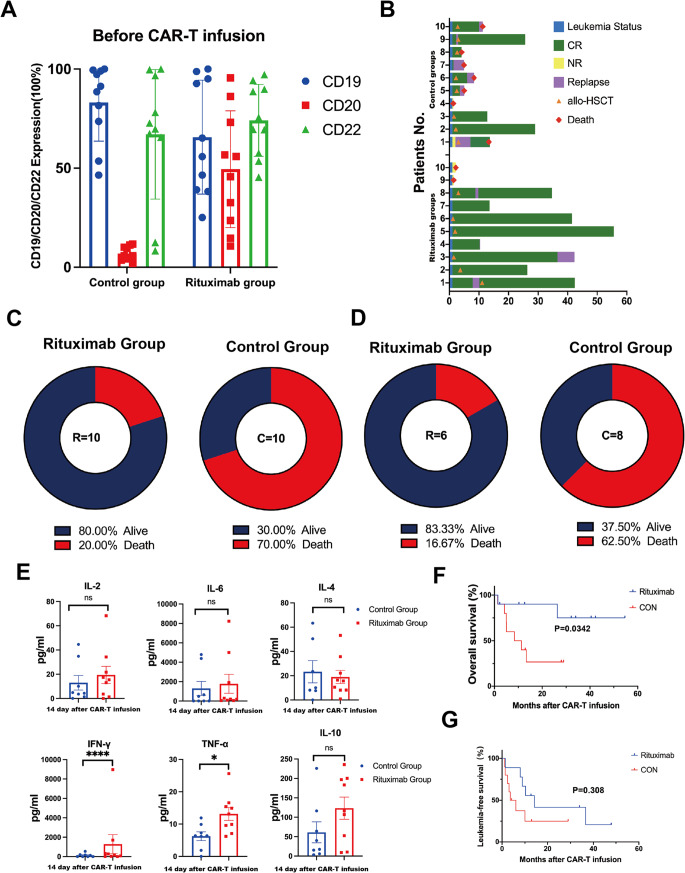
Clinical outcomes in the Rituximab combined with CAR-T and control groups. **A** CD19, CD20, and CD22 expression on the surface of leukemia cells in both groups of patients before receiving treatment. **B** Clinical outcomes, treatment response of each patient after Rituximab combined with CAR-T therapy and the duration of response. Patient number is shown to the left. **C** 6 of the 10 patient (60%) remained alive in the Rituximab group. 3 of the 10 patients (30%) remained alive in the Control group. **D** In the Rituximab group, 6 patients proceeded to allo-HSCT, of whom 5 (5/6, 83.3%) patients were alived with CR. In the Control group, 8 patients underwent allo-HSCT, of whom 3 (3/8, 37.5%) patients were alive with CR. **E** The peak levels of plasma cytokine release after CAR-T infusion. **F**, **G** Survival analysis. OS and LFS from the day of CAR-T cell infusion is shown for patients who received CAR-T combined with Rituximab (Rituximab, n = 10) compared with those who received CAR-T alone (CON, n = 10)

**Fig. 2:**
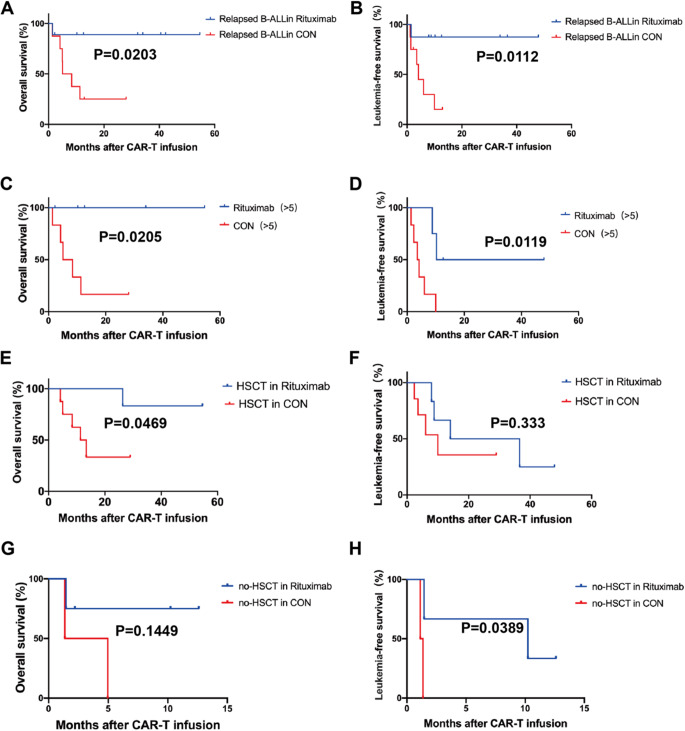
Effect of Rituximab combined with CAR-T in subgroups of patients. **A**, **B** OS and LFS in patients infused with CD19/CD22 CAR-T. (OS 66.7% vs 26.7%, *p* = 0.0927, LFS 66.7% vs 25.2%, *p* = 0.0314). **C**, **D** OS and LFS in relapsed patients. (OS 88.9% vs 25%, *p* = 0.0203, LFS 87.5% vs 15%, *p* = 0.0112). **E**, **F** The long-term prognosis in patients who had failed multiple lines of therapy (OS 100% vs 28.5%, *p* = 0.041; LFS 50% vs 0%, *p* = 0.004). **G**–**J** The long-term prognosis of patients whether or not they were bridged to transplantation after CAR-T treatment

**Fig. 3:**
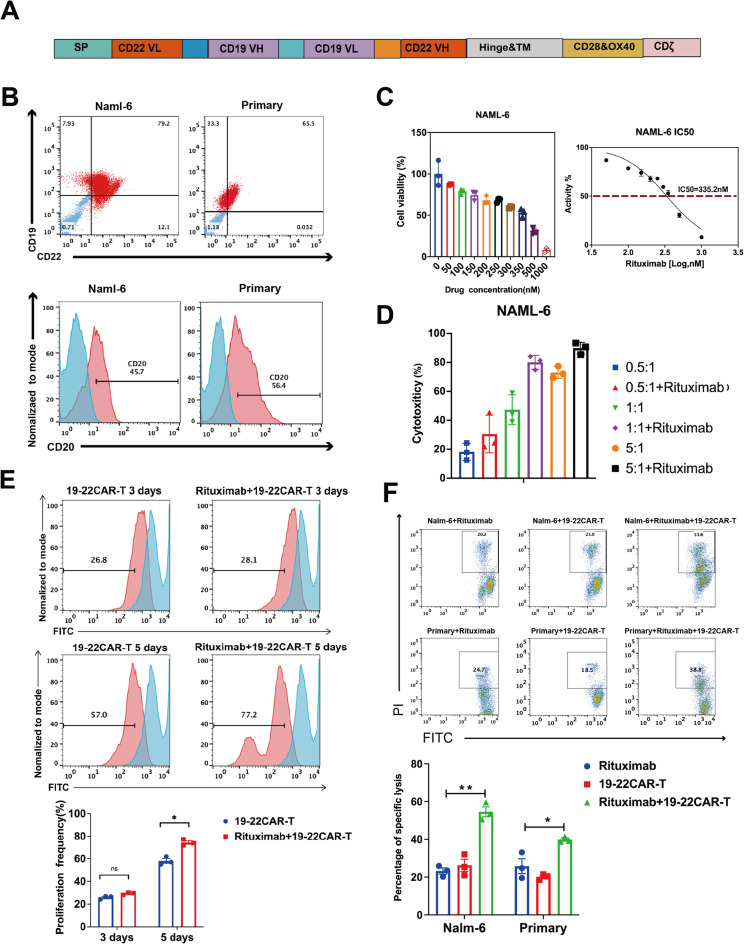
Expansion and Killing of CD19/CD22 CAR-T Cells was improved by Pre-treatment of Tumor Cells With Rituximab. **A** Schematic diagram of anti-CD19/CD22 CAR. **B** Expression of CD19, CD20 and CD22 antigens on NALM-6 as well as on primary cells. **C** The inhibition of NALM-6 by rituximab at different drug concentrations as measured by CCK8. Curves were plotted to calculate IC50 (335.2 nM). **D** Nalm-6 killing by rituximab combined with CAR-T was detected at different E/T ratio (0.5:1, 1:1, 5:1). **E** Upper panel:CAR T cells were labeled with CSFE and incubated with NALM-6 for 3–5 days. The proliferation of CD19/CD22 CAR-T cells was analyzed by flow cytometry. Lower panel: Histogram plots of the proliferation efficiency of CD19/CD22 CAR-T cells (n = 3 samples examined over three independent experiments). **F** Upper panel: CD19/CD22 CAR-T were co-incubated target cells (NALM-6, primary cells) at E/T ratios (1:1) for 24 h, and the target cells stained by PI were tested with Flow cytometry. Lower panel: Histogram plots of the apoptotic efficiency of CAR-T on target cells (NALM-6, primary cells) (n = 3 samples examined over three independent experiments)

### Lymphodepletion chemotherapy

Patients received lymphodepletion regimens: FC (Fludarabine 30 mg/m^2^/day and Cyclophosphamide 300 mg/m^2^/day) on days −5 to −3, with or without Decitabine (DAC) (total dose 100 mg/m^2^ in 3 days from day −6 to −4). The median dose of CAR-T cells was 1 × 10^7^ cells/kg (1–4 × 10^7^ cells/kg) during dose escalation.

### Clinical response assessment

All disease assessments were conducted per protocol or institutional practice. Response assessment was performed 28 days after CAR-T cell infusion. Complete remission (CR) was defined as less than 5% bone marrow (BM) blasts, the absence of circulating blasts, and no extramedullary sites of disease, regardless of cell count recovery. Complete response with incomplete count recovery was a complete response with cytopenia. MRD negativity was defined as less than 0.01% bone marrow blasts by flow cytometry. Relapsed disease was defined as the reappearance of blasts in the blood or bone marrow or in an extramedullary site after a complete remission.

### Toxicity assessment

Adverse events that occurred within one month after CAR-T therapy were recorded. Cytokine release syndrome (CRS) and immune effector cell-associated neurotoxicity syndrome (ICANS) were graded according to ASTCT Consensus Grading [6]. The diagnostic criteria for macrophage activation syndrome (MAS) were based on the MD Anderson diagnosis for patients post CAR-T cell therapy [7]. Other toxicities were assessed according to the National Cancer Institute Common Terminology Criteria for Adverse Events (CTCAE) version 5.0.

### Cell lines

The B lymphocyte leukemia cell line Nalm-6 was purchased from ATCC (Manassas, VA). Primary human ALL specimens were acquired from National Clinical Research Center for Hematologic Diseases. All cell lines were cultured in RPMI-1640 medium (Pricella, China)) supplemented with 10% foetal bovine serum (Sigma-Aldrich, USA), 2 mM L-glutamine (Pricella) and 100 U/mL peni-cillin/streptomycin (Pricella, China). This study design was approved by the First Affiliated Hospital of Soochow University Ethics Board. Written informed consent for publication of their clinical details was obtained from the patient/relative of the patient.

### In vitro functional assays

The proliferation of T cells was determined by CSFE staining in vitro. Individual groups of CAR T cells (1 × 10^6^ cells/tube) were labeled with CSFE (Beyotime, China) at 37 °C for 30 min. After being washed, individual groups of cells were stimulated with the same number of target cells that had previously been treated by mitomycin. CAR-T cells were collected after 5 days, and fluorescence was detected by flow cytometry.

To detect the optimized E:T ratio, CAR T cells were co-cultured with target cells at different E:T ratios (0.5:1, 1:1, 5:1) in 96-well plate. CCK8 was added to each well, and the optical density (OD) was measured after 4 h using Epoch (BioTek). The killing rate was calculated by the following equation: 1-([OD of sample]-[OD of negative control])/([OD of positive control]-[OD of negative control]).

For the apoptosis assay, CAR-T cells were incubated with target cells at E/T ratios (1:1) for 24 h, and the target cells stained by CSFE and PI were tested with Flow cytometry to evaluate the killing rate of effect cells. Effector cells and target cells were cultured at an E:T ratio of 1:1 in RPMI 1640 media for 24 h. Supernatant of culture was analyzed by Cytometric bead array according to the manufacturer’s instructions (BD Biosciences).

For the sequential killing assay, the numbers of residual CAR-T cells and Nalm-6 were quantified every 3 days using flow cytometry with counting beads, as previously described. Tumor cells were then added to the wells with CAR T cells to restore the original effector:target (E:T) ratio of 1:1. CCK8 is measured every 3 days to assess the ability of CAR-T to kill tumor cells.

The TUNEL assay was performed using the BrightRed Apoptosis Detection Kit (Vazyme, China), according to the manufacturer’s instructions. Images were obtained under a microscope (Nikon, Japan).

### Activation marker and exhaustion marker staining

CAR-T cells were co-cultured with Nalm-6 cells in the medium at the ratio of 1:1 E:T for 24, 48, and 72 h. The following anti-human antibodies were also used in this study: CD3 (Percp-cy5.5, 1:100 dilution), CD4 (FITC, 1:100 dilution), CD8 (APC, 1:100 dilution), CD25 (BB700, 1:100 dilution), CD69 (PE, 1:100 dilution), CD62L (PE, 1:100 dilution), PD-1 (PE, 1:100 dilution), TIM-3 (BB515, 1:100 dilution), LAG-3 (AF674, 1:100 dilution) were purchased from BD Biosciences. The resulting datas were analyzed by FlowJo software.

### Statistical analysis

OS was defined as the time from CAR-T cell infusion to death or the end of follow-up. LFS was defined as the time from CAR-T cell infusion to first relapse or death, the end of follow-up. OS and LFS were censored at the last follow-up. The probabilities of OS and LFS were estimated by the Kaplan-Meier (KM) method and assessed with the use of the log-rank test. Median follow-up time was estimated by using the reverse KM method. Categorical variables were performed with Fisher’s exact tests. Group comparisons were determined by two-tailed parametric t-tests for unpaired or paired data. All reported P values were two-sided, and statistical significance was defined as *P* < 0.05. All analyses were conducted using GraphPad Prism 8. The statistical test used for each figure is described in the corresponding figure legend.

## Result

### Patient characteristics

We retrospectively analyzed 20 r/r B-ALL patients who received CAR-T treatment between June 2017 and September 2021 at the First Affiliated Hospital of Soochow University. T Ten patients received Rituximab treatment before CAR-T cell infusion (Rituximab group), while the remaining ten patients did not receive rituximab treatment (Control group). The demographic and clinical characteristics of the r/r B-ALL patients are shown in Table 1, and there were no significant differences in baseline characteristics between the two groups. Both groups received treatment during the same period, ensuring no significant differences in supportive care (Table 1). Before treatment, the expression levels of CD19, CD20, and CD22 on the surface of leukemia cells were measured by flow cytometry in both groups (Rituximab group: CD19: 65.65%, CD20: 49.47%, CD22: 74.12%; Control group: CD19: 83.1%, CD20: 6.84%, CD22: 67.08%) (Fig. 1A).

**Table 1:** Patient characteristics

Patient characteristics	Rituximab group (n = 10)	Control group (n = 10)	*P* value
**Median age (range) years**	33 (7–58)	26 (7–56)	0.5681
**Sex no(%)**			>0.99
Female sex	7 (70%)	5 (50%)	
Male sex	3 (30%)	5 (50%)	
**Disease**			
B-ALL	10 (100%)	10 (100%)	>0.99
Refractory/relapsed	10 (100%)	10 (100%)	>0.99
**Number of recurrences before**			>0.99
**CAR-T, no.(%)**			
≤5	4 (40%)	3 (30%)	
>5	6 (60%)	7 (70%)	
**Extramedullary disease, no(%)**			0.6285
NO	6 (60%)	8 (80%)	
YES	4 (40%)	2 (20%)	
**Complex karyotype aberrations, no(%)**			>0.99
NO	7 (70%)	7 (70%)	
YES	3 (30%)	3 (30%)	
**PH**^**+**^**, no(%)**			>0.99
NO	8 (80%)	7 (70%)	
YES	2 (20%)	3 (30%)	
**Lymphodepletion regimens, no(%)**			>0.99
FC	8 (80%)	8 (80%)	
FC+DAC	2 (20%)	2 (20%)	
**CAR-T cell dose (×10**^**6**^**/kg), median (range)**	10 (4–20)	10 (1–20)	0.9016
**CAR-T cells origin**			>0.99
Patient	10 (100%)	9(90%)	
Donor	0	1 (10%)	
**CRS grade**			0.814
CRS grade 0	1 (10%)	2 (20%)	
CRS grade 1–2	7 (70%)	6 (60%)	
CRS grade 3 (hematological toxicity)	2 (20%)	2 (20%)	
**CAR-T response**			0.6285
MRD-CR	2 (20%)	4 (40%)	
MRD+CR	8 (80%)	6 (60%)	
**Bridging therapy (HSCT)**			>0.99
No	3 (30%)	3 (30%)	
Yes	7 (70%)	7 (70%)	

### Clinical response

28 days after CAR-T cells infusion, 8 out of 10 patients (80%) in the Rituximab group achieved minimal residual disease negative complete remission (MRD-negative CR), maintaining CR for a median time of 11.15 months (0.46–46.93) post-CAR-T therapy. In comparison, 6 out of 10 patients (60%) in the Control group achieved MRD-negative CR, maintaining CR for a median time of 5.03 months (0.13–28) post-CAR-T therapy (*p* = 0.158) (Table 1, Fig. 1B). At the time of analysis, 8 out of 10 patients (80%) remained alive in the Rituximab group, while 3 out of 10 patients (30%) remained alive in the Control group (Fig. 1C). Among the patients in the Rituximab group, 6 (60%) proceeded to allo-HSCT, with 5 out of 6 (83.3%) patients achieving CR and survival. In contrast, 8 patients (80%) in the Control group underwent allo-HSCT, but only 3 out of 8 (37.5%) patients were alive with CR (Fig. 1D). Immune-modulating cytokines, including TNF-α, IFN-γ, IL-2, IL-6, IL-4, and IL-10, were induced in patients following 19–22 CAR-T and rituximab infusion, showing more significant elevation in the Rituximab Group compared to the Control Group (Fig. 1E).

### Long-term survival

The median follow-up periods for the Rituximab and Control groups were 29.27 and 9.83 months, respectively. Our findings suggest that adding Rituximab may lead to a more favorable prognosis compared to the Control group. The 2-year overall survival (OS) and leukemia-free survival (LFS) rates were both higher in the Rituximab group compared to the Control group (90% vs 26.7%, *p* = 0.0342; 41.7% vs 25%, *p* = 0.308) (Fig. 1G). Subgroup analysis revealed that Rituximab significantly improved OS and LFS for relapsed patients (88.9% vs 25%, *p* = 0.0203; 87.5% vs 15%, *p* = 0.0112) (Fig. 2A, B). Furthermore, the combination of CAR-T and Rituximab improved long-term prognosis in patients who had failed multiple lines of therapy (>5) (OS 100% vs 28.5%, *p* = 0.0205; LFS 50% vs 0%, *p* = 0.0119) (Fig. 2C, D). Following CAR-T treatment, subgroups of patients who underwent bridging allo-HSCT or not were analyzed. The long-term prognosis of the Rituximab group was found to be superior to that of the Control group. For patients who received allo-HSCT after CAR-T therapy, OS in the Rituximab group was significantly longer than in the Control group (83.3% vs 33.3%, *p* = 0.0469) (Fig. 2E, F). For patients who did not undergo allo-HSCT after CAR-T therapy, LFS in the Rituximab group was significantly better than in the Control group (33.3% vs 0%, *p* = 0.0389) (Fig. 2G, H).

### Safety

There were no significant differences between the two groups in terms of hematological and non-hematological toxicities. All patients experienced grade 1–3 cytokine release syndrome (CRS) symptoms, such as fever and hypotension, which were effectively managed through symptomatic treatment. Two patients in the Rituximab group developed headaches, while patients in the Control group did not exhibit any neurotoxicity syndromes. Moreover, all patients in our study experienced hematological toxicities. The median time to platelet and neutrophil count recovery was similar in both groups. Additionally, 50% (5/10) of patients in the Rituximab group experienced infections, compared to 30% (3/10) in the control group. All adverse events were reversible and manageable (Table 1).

### Expansion and killing of CD19/CD22 CAR-T cells was improved by pre-treatment of tumor cells with Rituximab

Our clinical data indicated that Rituximab combined with CD19/CD22 CAR-T treatment effectively improved the long-term prognosis of r/r B-ALL patients (Fig. 3A). Subsequently, we evaluated the killing ability of Rituximab combined with CAR-T on tumor cells and the proliferation of CAR-T in vitro.

Firstly, we evaluated the tumor killing effect by selecting CD19, CD20, and CD22 simultaneously antigen-expressing B-ALL cells (NALM-6) as well as primary cells (Fig. 3B). Subsequently, we identified the concentration of Rituximab in vitro that was effective for killing tumor cells (NALM-6, Primary cells) without impacting CAR-T activity (Fig. 3C, Supplementary 1A). We used a concentration of 350 nM Rituximab to treat tumor cells, followed by flow cytometry to detect the expression of CD19 and CD22. The results showed that the expression of CD19 and CD22 had not been obviously altered (Supplementary 1C). We also selected an efficacy target ratio based on the killing of NALM-6 by Rituximab combined with CAR-T cells, and eventually, we chose a 1:1 efficacy target ratio for the subsequent experiments (Fig. 3D).

In proliferation experiments, we found CD19-CD22 CAR-T cells rapidly proliferated upon Nalm-6 challenge, reaching a peak level of expansion on day 5 (Fig. 3E). Rituximab presensitized tumor cells facilitated the proliferation of CAR-T cells.

Several mechanisms could be working through the therapeutic efficacies of anti-CD20 antibodies, including complement-dependent cytotoxicity (CDC), antibody-dependent cellular cytotoxicity (ADCC) and the induction of apoptosis [8]. Previous studies have indicated that direct effects mediated through binding of CD20 to the cell surface include inhibition of proliferation, induction of apoptosis, and sensitization of cancer cells to chemotherapy [9]. We divided mitomycin-treated NALM-6 and primary cells into two groups based on whether they were pretreated with Rituximab or not, and then incubated them with CD19/CD22 CAR-T cells. The other group was treated with Rituximab alone for tumor cells. FCM analysis showed that Rituximab combined with CAR-T significantly promoted tumor cell apoptosis compared to the control groups (*p* < 0.001). Terminal deoxynucleotidyl transferase dUTP nick end labeling (TUNEL) assays demonstrated that the number of apoptotic cells in the Rituximab combined with CAR-T group was significantly higher than that in the control groups (Fig. 3F, Supplementary 1D).

We utilized a sequential killing assay in which CAR-T cells were plated with Nalm-6 cells at a 1:1 effector-to-target ratio and replated every 3 days with fresh tumor cells to restore the initial ratio. Unlike the control groups, the combination of Rituximab with CAR-T repeatedly eliminated tumor cells for at least three rounds (Supplementary 2A). Collectively, the Rituximab combined with CAR-T demonstrated robust and sustained cytotoxicity against ALL cell lines in vitro.

**Fig. 4:**
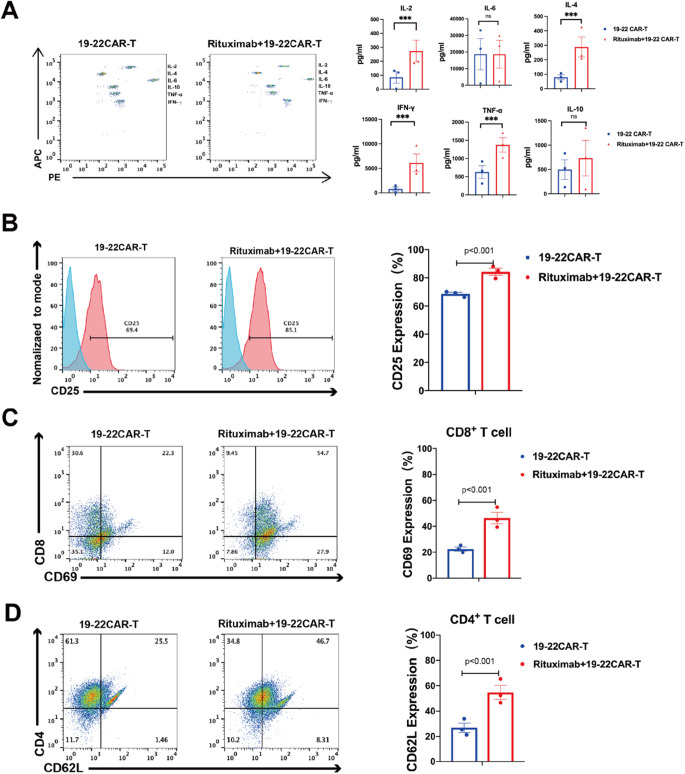
CD19/CD22 CAR-T cells undergo activation, antigen-induced differentiation. **A** CD19/CD22 CAR-T cells produced higher levels of cytokines. CAR-T cells and T cells were incubated with tumor cells for 12 h; the levels of cytokines in the culture supernatants were determined by ELISA assay. **B**–**D** Left panel: CD19/CD22 CAR-T were incubated with NALM-6 for 48 h, CD25, CD8+CD69, CD4+CD62L were analyzed by flow cytometry. Quantitative analysis of the frequency of CD25, CD8+CD69, CD4+CD62L; Right panel: Histogram plots of CD25, CD8+CD69, and CD4+CD62L expression on CD19/CD22 CAR T cells (n = 3 samples examined over three independent experiments)

To examine the effector function of rituximab combined with CAR-T, a panel of cytokines were measured during the in vitro cytotoxicity assay. Compared with the control group, a broad range of cytokines was produced by Rituximab combined with CD19-CD22 CAR-T when co-cultured with NALM-6. Increased secretion of effector cytokines and chemokines such as interferon-γ (IFN-γ), tumor necrosis factor-α (TNF-α), and interleukin-2 (IL-2) was observed (Fig. 4A).

### Pre-treatment of tumor cells with Rituximab affects the surface molecule expression of CD19-CD22 CAR-T

To investigate the effects of Rituximab on the expression of surface marker molecules on CD19/CD22 CAR-T cells, we co-cultured Rituximab-treated tumor cells with CAR-T cells for 24 h. Subsequently, we assessed the expression of activation molecules and memory molecules on CAR-T cells using flow cytometry. The analysis revealed that CD25+, CD8+CD69+, and CD4+CD62L+ molecules on the surface of CD19/CD22 CAR-T cells were significantly upregulated in the Rituximab pre-treatment group compared to the group without Rituximab treatment (*p* < 0.001) (Fig. 4B–D).

**Fig. 5:**
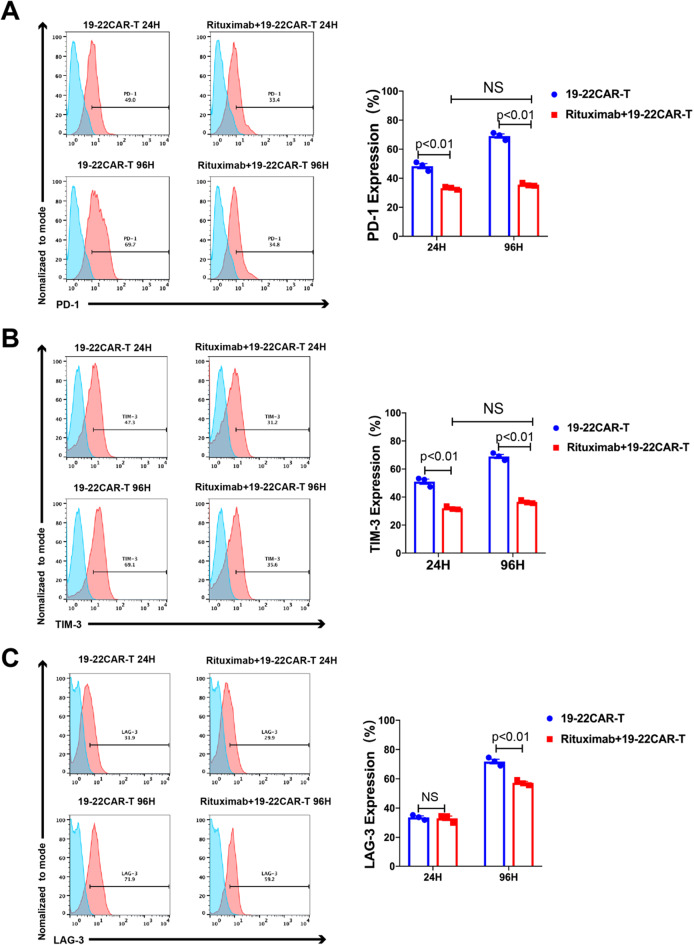
Rituximab reduced the expression of exhaustion molecules (PD-1, LAG-3, Tim-3) on CD19/CD22 CAR-T. **A**–**C** Left panel: CD19/CD22 CAR-T were incubated with NALM-6 for 24–96 h, PD-1, LAG-3, and Tim-3 were analyzed by flow cytometry. Quantitative analysis of the frequency of PD-1, LAG-3, and Tim-3; Right panel: Histogram plots of PD-1, LAG-3, and Tim-3 expression on CD19/CD22 CAR T cells (n = 3 samples examined over three independent experiments)

To investigate the influence of Rituximab on the persistence of CD19/CD22 CAR-T cells, the expression of inhibitory molecules PD-1, LAG-3 and Tim-3 on CD19/CD22 CAR-T cells were monitored. Flow cytometry (FCM) analysis at 24 h showed that the expression of CD19/CD22 CAR-T cell surface exhaustion molecules (LAG-3, PD-1, TIM-3) was significantly lower in the Rituximab group than in the Control group (*p* < 0.001). Interestingly, compared to the control group, FCM analysis at 96 h showed that pretreatment of tumor cells with rituximab did not significantly upregulate CD19/CD22 CAR-T surface exhaustion molecules (PD-1, TIM-3) (Fig. 5A–C).

To establish whether Rituximab combined with CAR-T was resistant to functional exhaustion, we used a sequential killing assay in which CAR-T cells were plated with Nalm-6 cells at a 1:1 effector-to-target ratio and replated every 3 days with fresh tumor cells to restore the initial ratio. After three rounds of repeated challenges, Rituximab combined with CAR-T effectively reduced CAR-T exhaustion (Supplementary 2B–D).

## Discussion

CAR-T cells have emerged as a potent therapeutic weapon against B cell hematologic malignancies, leading to an exponential increase in clinical trials [10]. Despite the high success rate in treating ALL, there is still a 40% recurrence rate. The strong activation during clinical therapy drives CAR-T cell exhaustion and even apoptosis, which limits their antitumor efficacy [11, 12]. A Long-Term Follow-up study of CD19 CAR-T Therapy revealed that patients with a low disease burden had a significantly longer event-free survival (EFS) and overall survival (OS) compared to those with a high disease burden [1]. Therefore, reducing tumor burden prior to CAR-T therapy could be a strategy with beneficial effects on the long-term survival of relapsed/refractory B-ALL.

Rituximab has revolutionized the treatment of B-cell malignancies, being part of the standard of care for FL [13], DLBCL [14], and CLL [15]. Recent clinical studies have shown that Rituximab also exhibits good antitumor effects in other B lineage hematologic tumors. Clinical trials have demonstrated that Rituximab combined with chemotherapy can increase event-free survival (EFS) compared to chemotherapy alone in Philadelphia chromosome-negative acute lymphoblastic leukemia [16–18]. Thus, we successfully treated relapsed/refractory (r/r) B-ALL patients who had failed multiple lines of therapy with Rituximab combined with CAR-T. Our clinical data showed that Rituximab combined with CAR-T improved the long-term prognosis of r/r B-ALL patients, regardless of whether they were bridged to transplantation after CAR-T treatment. Additionally, for patients infused with CD19/CD22 CAR-T cells, Rituximab combined with CAR-T effectively improved the leukemia-free survival (LFS) of r/r B-ALL patients. We observed that pretreatment of tumor cells with Rituximab enhanced the expansion of CD19/CD22 CAR-T cells in vitro. Concurrently, flow cytometry (FCM) analysis showed significant upregulation of CD25+, CD8+CD69, and CD4+CD62L+ on the surface of CD19/CD22 CAR-T cells (*p* < 0.001).

Subsequently, in Cytotoxicity Assays, we demonstrated that Rituximab pretreatment of tumor cells increased the killing activity of CAR-T cells. Our data in vitro demonstrated that Rituximab efficiently reduced tumor burden, and the combination of CAR-T with Rituximab enhanced the killing efficiency of CAR-T in vitro. Accordingly, we conclude that Rituximab might enhance the efficacy of CAR-T cells by reducing tumor burden.

In a subgroup analysis, we found that Rituximab combined with CAR-T effectively improved the long-term prognosis of relapsed patients. Furthermore, Rituximab combined with CAR-T significantly improved leukemia-free survival (LFS) in patients who had failed multiple lines of therapy, suggesting that Rituximab may be effective in relapsed patients, particularly those who have failed multiple lines of therapy. In relapsed patients, CAR-T cells are depleted due to repetitive antigen stimulation, as evidenced by the upregulation of CAR-T exhaustion molecules and dysregulation of activation molecules [18, 19]. Nevertheless, there was limited evidence on whether Rituximab was an effective approach to reduce CAR-T exhaustion. T To investigate whether Rituximab has an effect on CAR-T persistence, we stimulated CAR-T cells in vitro with Rituximab-treated Nalm-6 cells to evaluate the changes in CAR-T surface exhaustion molecules at different times. We found that the expression of CAR-T cell surface exhaustion molecules (LAG-3, PD-1, TIM-3) was significantly lower in the Rituximab group than in the control group. Additionally, the upregulation of CAR-T cell surface exhaustion molecules was significantly slower in the Rituximab group compared to the control group.

In the sequential killing assay, we observed that Rituximab combined with CAR-T demonstrated robust and sustained cytotoxicity against ALL cell lines in vitro. After three rounds of repeated challenges, Rituximab combined with CAR-T effectively reduced CAR-T exhaustion. Therefore, we speculated that Rituximab combined with CAR-T treatment could potentially enhance CAR-T efficacy by reducing its exhaustion. In terms of safety, the combination of rituximab with CAR-T therapy did not induce higher hematological and non-hematological toxicities.

We hypothesize that these effects may be attributed to changes in apoptotic products of tumor cells induced by rituximab pretreatment. These apoptotic products stimulate CAR-T cells to enhance their tumor-killing efficacy, resulting in a significant upregulation of exhaustion molecules and memory phenotypes in CAR-T cells. Subsequently, we are prepared to conduct metabolomics analysis to further explore the mechanism by which rituximab enhances the functionality of CAR-T cells.

To the best of our knowledge, this is the first report assessing the efficacy and safety of rituximab combined with CAR-T therapy in patients with relapsed or refractory B-ALL. This study represented the successful application of rituximab combined with CAR-T therapy in patients with r/r B-ALL who have failed multiple lines of therapy. In our study, rituximab combined with CAR-T therapy showed an excellent safety record with manageable adverse effects in patients. Additionally, we conducted in vitro assays to investigate how rituximab enhances CAR-T capabilities. Despite the limitations of the data, our study pioneers the observation of the clinical benefits associated with rituximab combined with CAR-T therapy and further investigates the effect of rituximab on CAR-T cells in vitro, partially illustrating the effectiveness of this combination.

Currently, CAR-T therapy for hematologic cancer has a remarkable outcome, however, there are still challenges to be overcome. Using monoclonal antibody in combination with CAR-T therapy is an approach which is effective and convenient in the clinical condition, thus we can broaden the application to other hematologic cancers, such as acute myeloid leukemia, in order to improve the CAR-T efficiency in treating acute myeloid leukemia.

## Electronic supplementary material

Below is the link to the electronic supplementary material.

Supplementary Material 1

Supplementary Material 2

## Data Availability

All data generated or analyzed during this study, together with the Supplementary files, are included in this published article. Meanwhile, the datasets involved in the current study are available from the corresponding author on reasonable request.
