# Supplementary figures and images for: Rituximab potentially improves clinical outcomes of CAR-T therapy for r/r B-ALL via sensitizing leukemia cells to CAR-T-mediated cytotoxicity and reducing CAR-T exhaustion
Source: Cell Oncol (Dordr). 2024 Apr 25;47(5):1649–61. doi: 10.1007/s13402-024-00945-7 (PMC11467070; doi:10.1007/s13402-024-00945-7)

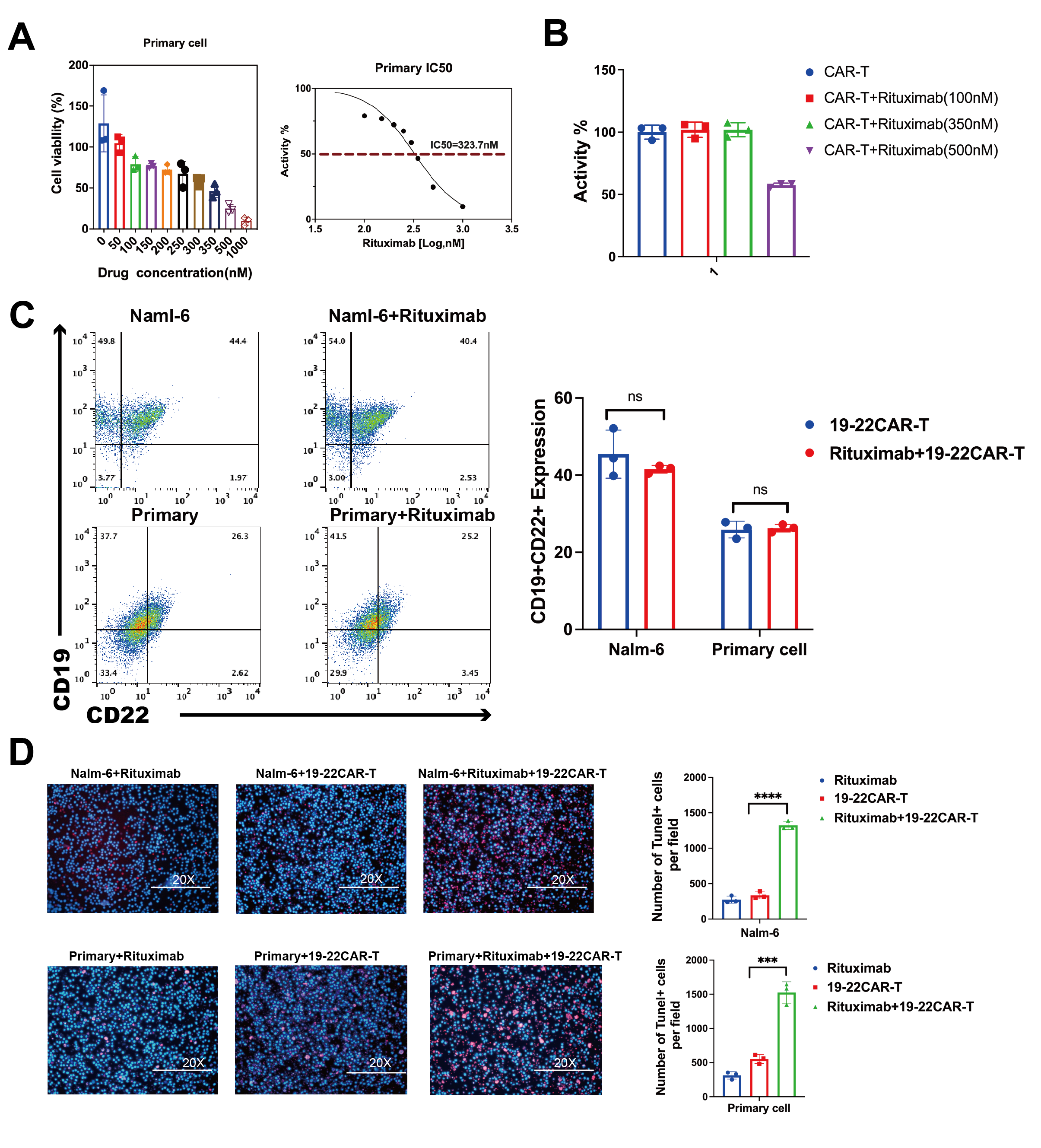

Supplement: Supplementary file 1 — Supplementary Material 1 [file 13402_2024_945_MOESM1_ESM.png]

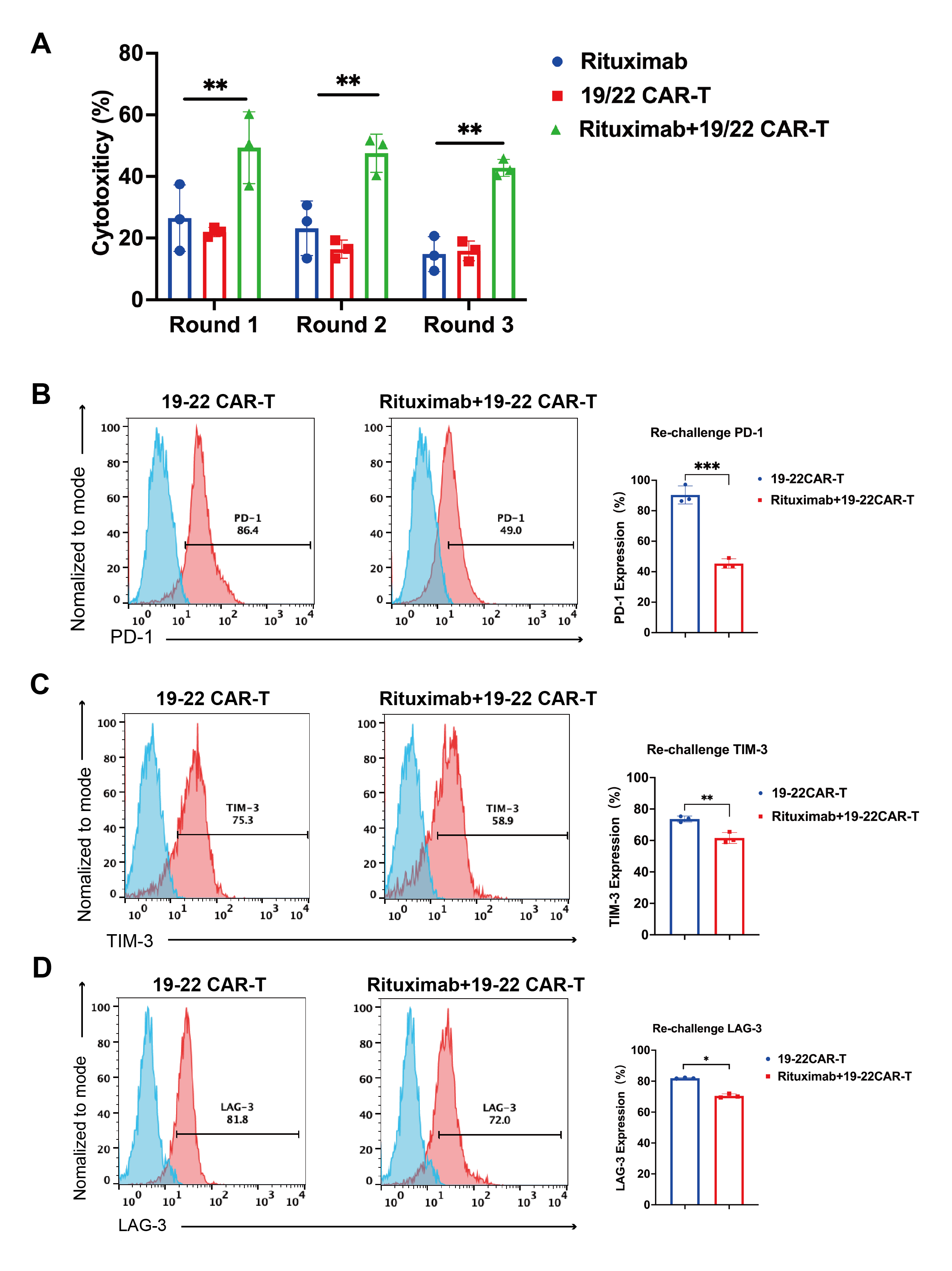

Supplement: Supplementary file 2 — Supplementary Material 2 [file 13402_2024_945_MOESM2_ESM.png]
